# RPPA-based protein profiling reveals eIF4G overexpression and 4E-BP1 serine 65 phosphorylation as molecular events that correspond with a pro-survival phenotype in chronic lymphocytic leukemia

**DOI:** 10.18632/oncotarget.4104

**Published:** 2015-05-12

**Authors:** Austin Y. Shull, Satish K. Noonepalle, Farrukh T. Awan, Jimei Liu, Lirong Pei, Roni J. Bollag, Huda Salman, Zhiyong Ding, Huidong Shi

**Affiliations:** ^1^ Department of Biochemistry & Molecular Biology, Georgia Regents University, Augusta, Georgia, USA; ^2^ GRU Cancer Center, Georgia Regents University, Augusta, Georgia, USA; ^3^ The Ohio State Comprehensive Cancer Center, The Ohio State University, Columbus, Ohio, USA; ^4^ Department of Pathology, Georgia Regents University, Augusta, Georgia, USA; ^5^ Deparment of Medicine, Georgia Regents University, Augusta, Georgia, USA; ^6^ Department of Systems Biology, University of Texas MD Anderson Cancer Center, Houston, Texas, USA

**Keywords:** CLL, RPPA, EIF4G, 4E-BP1, NVP-BEZ235

## Abstract

Chronic lymphocytic leukemia (CLL), the most common adult leukemia, remains incurable despite advancements in treatment regimens over the past decade. Several expression profile studies have been pursued to better understand CLL pathogenesis. However, these large-scale studies only provide information at the transcriptional level. To better comprehend the differential protein changes that take place in CLL, we performed a reverse-phase protein array (RPPA) analysis using 167 different antibodies on B-cell lysates from 18 CLL patients and 6 normal donors. From our analysis, we discovered an enrichment of protein alterations involved with mRNA translation, specifically upregulation of the translation initiator eIF4G and phosphorylation of the cap-dependent translation inhibitor 4E-BP1 at serine 65. Interestingly, 4E-BP1 phosphorylation occurred independently of AKT phosphorylation, suggesting a disconnect between PI3K/AKT pathway activation and 4E-BP1 phosphorylation. Based on these results, we treated primary CLL samples with NVP-BEZ235, a PI3K/mTOR dual inhibitor, and compared its apoptotic-inducing potential against the BTK inhibitor Ibrutinib and the PI3Kδ inhibitor Idelalisib. We demonstrated that treatment with NVP-BEZ235 caused greater apoptosis, greater apoptotic cleavage of eIF4G, and greater dephosphorylation of 4E-BP1 in primary CLL cells. Taken together, these results highlight the potential dependence of eIF4G overexpression and 4E-BP1 phosphorylation in CLL survival.

## INTRODUCTION

Chronic lymphocytic leukemia (CLL) is a malignant disease characterized by the accumulation of monoclonal B cells that have escaped their regulated life cycle. CLL is the most common adult leukemia in the Western world, and though it allows longer survival than most leukemias, this disease creates a poor quality of life and currently remains incurable [1]. Particular factors that contribute to the incurability of CLL are the inter- and intra-tumor heterogeneity that exists among CLL populations and the subclonal expansion of refractory CLL cells that can arise after initial chemotherapy treatment [2, 3]. Based on these underlying characteristics of CLL, many large-scale biomarker studies have focused on determining molecular features that would help provide potential predictive and prognostic power in treating this disease. These studies have typically relied on mRNA-based gene expression or DNA-based somatic mutation and CpG methylation analysis. Such studies have created the opportunity to discover genetic and epigenetic marks as potential prognostic tools. Examples of these discovered prognostic markers include somatic mutations in genes like *SF3B1*, *NOTCH1*, and *MYD88* [4-6], as well as differential gene expression and DNA methylation changes in genes like *ZAP70*, *LPL*, *CRY1*, and *LDOC1* [7-14]. Along with discovering these single gene prognostic markers in CLL, genome-wide DNA methylation and gene expression studies have also demonstrated genomic signatures that correspond with specific CLL subtypes, like IGVH mutation and *CD38* expression status and have provided better comprehension of the molecular abnormalities that occur in this disease [3, 8, 11, 12, 15-17]. Nevertheless, though the understanding of CLL pathobiology has been greatly enhanced by these large-scale gene studies, the limiting aspect that still remains in RNA or DNA-based profiling is the inability to provide clarity to the altered protein expression and signaling landscape in CLL.

Understanding how pro-proliferative proteins are differentially altered in CLL has currently gained popularity based on recent studies that demonstrate the efficacy of targeting signaling proteins involved in the B-cell Receptor (BCR) pathway [18-22]. Most of these efficacious effects observed when targeting the BCR pathway have stemmed from the selective inhibition of either the Bruton's Tyrosine Kinase (BTK) using the inhibitor Ibrutinib or the PI3K-delta (PI3Kδ) specific kinase using the inhibitor Idelalisib. Based on the recent success of these two inhibitors' respective clinical trials, the outlook of treatment strategies for CLL has changed and motivation has shifted towards better understanding the specific protein events that drive CLL pathogenesis [23-27].

Though several proteins have individually been investigated when determining the oncogenic features of CLL, no current study has simultaneously examined numerous protein alterations on a comprehensive level. Based on this current void, our goal was to perform reverse-phase protein array (RPPA) analysis using 167 antibody probes on primary CLL lysates in order to determine the deregulated protein events that occur in CLL. From this study, we demonstrate that the AKT/mTOR-related proteins are altered in CLL, with significant alteration occurring in the downstream mRNA translational machinery proteins eukaryotic translation factor 4G (eIF4G) and the eukaryotic translation initiation factor 4E-binding protein 1 (4E-BP1). These results and corresponding *ex vivo* treatment results with the dual PI3K/mTOR inhibitor NVP-BEZ235 help demonstrate the potential dependence of mRNA translation in CLL survival, as well as revealing mRNA translation as a potential therapeutic target in CLL.

## RESULTS

### Comprehensive analysis of RPPA probe intensities reveals a common protein signature among CLL patients

**Figure 1:**
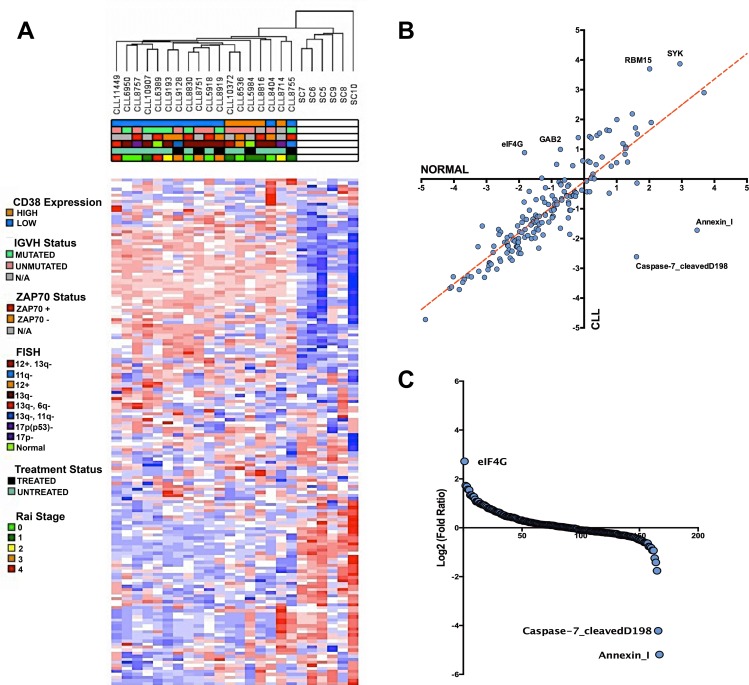
Altered protein landscape common among CLL patients **A.** Non-supervised hierarchical clustering of normalized RPPA probe intensities using Euclidean distance. Heatmap demonstrates the separation of the CLL protein landscape and normal B-cell landscape without any statistical separation. Based on the clinical parameters, the altered protein expression is generally ubiquitous among CLL patients. **B.** Scatter plot comparing the average intensity of all 167 probes in CLL versus the average probe intensity in healthy B-cells **C.** Rank ordering plot based on the transformed (log2) fold ratio between CLL expression and normal B-cell expression. Rank ordering plot demonstrates the contrast between eIF4G upregulation, diminished cleaved caspase-7, and Annexin I downregulation from other probes in the RPPA dataset.

To gain a better understanding of the expression profile of CLL at the protein level, we collected 18 CLL patient and 6 healthy CD19+ B-cell lysates for RPPA analysis. The CLL lysates collected for this study are comprised of samples containing varying IGVH mutation, *CD38* expression, FISH, *ZAP70* expression, treatment, and Rai stage status in order to determine whether these clinical parameters demonstrate any large-scale protein signatures (Table 1). From the collected array data, we gained an initial understanding of how the clinical features overlapped with the corresponding molecular signature determined by non-supervised hierarchical clustering. Based on the clustered dendrogram and the heatmap patterns, there seems to be a clear separation in molecular profiles between CLL patients and healthy donors samples, demonstrating an overall transformation at the protein level between the cancer and normal phenotype. However, the contrast between CLL patient subtypes is less stark as there seems to be a less clear separation within the unsupervised hierarchical clustering patterns of the clinical subfeatures ascribed to the patient samples (Figure 1A). Thus, unsupervised clustering would suggest that an underlying protein signature that distinguishes itself from normal CD19+ B-cells is common among CLL patient samples.

**Table 1:** Clinical characteristics of CLL patient samples analyzed using reverse-phase protein array (RPPA)

Sample ID	IGVH Status	CD38 Expression	ZAP70 Status	11q Deletion	13q Deletion	17p Deletion	Trisomy 12	Treatment	Rai Stage
CLL5918	UNMUTATED	LOW	ZAP70+	NORMAL	13q-	NORMAL	NORMAL	UNTREATED	0
CLL5984	UNMUTATED	HIGH	N/A	NORMAL	NORMAL	NORMAL	NORMAL	TREATED	1
CLL6389	MUTATED	LOW	ZAP70+	NORMAL	NORMAL	NORMAL	NORMAL	UNTREATED	4
CLL6536	UNMUTATED	HIGH	ZAP70-	NORMAL	NORMAL	NORMAL	12+	UNTREATED	0
CLL6950	MUTATED	LOW	N/A	NORMAL	13q-	NORMAL	NORMAL	UNTREATED	0
CLL8404	UNMUTATED	LOW	ZAP70-	NORMAL	13q-	NORMAL	NORMAL	UNTREATED	0
CLL8714	N/A	HIGH	N/A	NORMAL	NORMAL	17p-	NORMAL	UNTREATED	2
CLL8751	UNMUTATED	LOW	N/A	NORMAL	13q-	NORMAL	NORMAL	TREATED	3
CLL8755	MUTATED	LOW	ZAP70+	11q-	NORMAL	NORMAL	NORMAL	TREATED	1
CLL8757	UNMUTATED	LOW	ZAP70+	NORMAL	NORMAL	17p-	NORMAL	UNTREATED	0
CLL8816	N/A	HIGH	ZAP70+	NORMAL	13q-	NORMAL	NORMAL	UNTREATED	1
CLL8830	MUTATED	LOW	ZAP70+	NORMAL	13q-	NORMAL	NORMAL	UNTREATED	0
CLL8919	MUTATED	LOW	ZAP70-	NORMAL	NORMAL	NORMAL	NORMAL	TREATED	3
CLL9128	UNMUTATED	LOW	ZAP70-	11q-	13q-	NORMAL	NORMAL	TREATED	3
CLL9193	MUTATED	LOW	ZAP70-	NORMAL	13q-	NORMAL	NORMAL	UNTREATED	2
CLL10372	UNMUTATED	HIGH	N/A	NORMAL	13q-	NORMAL	12+	UNTREATED	1
CLL10907	MUTATED	LOW	N/A	NORMAL	13q-	NORMAL	NORMAL	UNTREATED	1
CLL11449	UNMUTATED	LOW	N/A	NORMAL	13q-	NORMAL	NORMAL	UNTREATED	4

By further investigating the RPPA intensities of the antibody probes, we were able to determine through correlation comparison that eIF4G, SYK, RBM15, and GAB2 expression is higher in CLL than in healthy donor B-cells, whereas Annexin I and cleaved caspase 7 is lower in CLL compared to healthy donors (Figure 1B). In fact, based on log-fold/rank comparison of all probes represented in the RPPA panel, eIF4G overexpression is the most upregulated protein event in our CLL cohorts, whereas the pro-apoptotic protein events Annexin I expression and cleaved-caspase 7 are the most downregulated events in CLL [28-31] (Figure 1C). These extreme protein expression changes are examples that highlight the aberrant changes within the CLL protein signature.

### Supervised protein & pathway analysis in CLL reveals upregulation of mRNA translation machinery proteins

**Figure 2:**
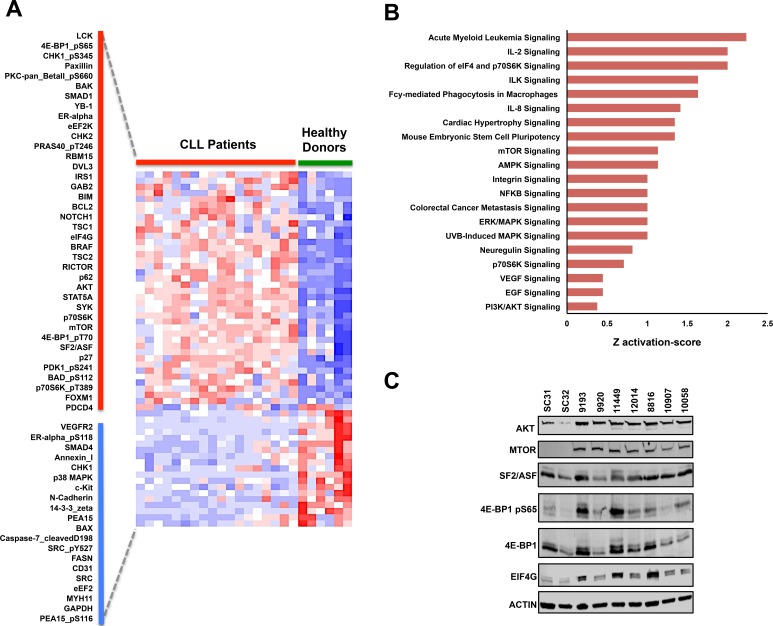
Supervised RPPA analysis reveals potential oncoproteins in CLL **A.** Supervised hierarchical clustering analysis of RPPA results using an ANOVA FDR *p*-value threshold lower than 0.05 and a fold difference threshold of greater than 1.25 and less than −1.25. Based on this threshold, 58 probes were differentially altered in CLL with 38 probes being upregulated (along red bar) and 20 probes being downregulated (along blue bar). **B.** Ontology analysis of the differently expressed probes using Ingenuity Pathway Analysis (IPA) **C.** Immunoblot validation of differentially modified proteins that are involved in mRNA translation regulation.

To systematically investigate the specific protein events that significantly correspond with the CLL phenotype, we further analyzed the protein arrays using ANOVA to determine which antibodies had significant differential probe intensities between CLL and normal B-cells samples. Based on this analysis, we determined that 58 distinct antibody probe intensities separate the CLL phenotype from the normal B-cell phenotype (Fold change > 1.25 or < −1.25, FDR *p*-value<0.05) with 38 probes being upregulated and 20 being downregulated in CLL (Figure 2A). The differentially altered proteins and phosphoproteins identified include proteins that have previously been associated with CLL, specifically the SYK and LCK kinase proteins and the Notch-1 signaling activator [32-41]. Paradoxically, we also identified the pro-apoptotic proteins BIM and BAK to be upregulated in our RPPA dataset. Nevertheless, the protein profile demonstrated by RPPA analysis helps provide a larger frame of reference for the molecular pathology of CLL as BIM overexpression in CLL commonly correlates with the overexpression of its direct anti-apoptotic antagonist BCL2, thus counteracting pro-apoptotic function of BIM [36]. A similar counteraction also occurs for the pro-apoptotic functions of BAK due to the fact that its pro-apoptotic binding partner BAX [42-44] is significantly downregulated in CLL patients. By identifying protein expression patterns of BIM, BCL2, BAK, and BAX as well as identifying the phosphorylation of the pro-apoptotic BAD at serine 112 (Table 2; Figure 2A), RPPA analysis helps provide a comprehensive illustration of how the anti-apoptotic characteristics of CLL are mediated among the BCL2 protein family.

**Table 2:** Expression profile of BH3-family proteins in CLL RPPA dataset

Protein	Apoptotic	CLL Fold Ratio
Bim	Pro	2.53
Bcl-2	Anti	2.52
Bak	Pro	1.47
Bad_pS112	Anti	1.34
Mcl1	Pro/Anti	1.08
Bcl-xL	Anti	1.03
Bid	Pro	−1.22
Bax	Pro	−1.26

Supervised hierarchical analysis using ANOVA further revealed differential expression of several proteins implicated in cancer, including BRAF, STAT5A, and the Wnt pathway-related protein DVL3 [45-48]. Nonetheless, several of the proteins that were differentially altered in our array dataset are specifically involved in the AKT/mTOR signaling pathway [49, 50]. This signaling cascade is highly important in several types of cancers due to the fact that AKT and mTOR can regulate numerous tumorigenic events including cell proliferation, cell cycle progression, apoptotic resistance, autophagy, nutrient-dependent growth, and protein translation initiation [51-58]. Interestingly, the expression of total AKT and total mTOR were both significantly upregulated in CLL compared to healthy donor B-cells. These two events observed in our CLL samples are specifically noteworthy as upregulation of other kinases at the protein level have previously been identified in CLL, including the critical LCK, SYK, and BTK kinase proteins [23, 32, 35]. We also identified overexpression of scaffolding proteins that are traditionally involved in the AKT/mTOR pathway including IRS1, RICTOR, and GAB2, which have been implicated as oncogenic contributors in other cancer types [59-65]. With overexpression of these AKT/mTOR-related signaling proteins, we also observed higher phosphoprotein levels for PDK1 at serine 241, 4E-BP1 at serine 65 and threonine 70, p70S6K at threonine 389, BAD at serine 112, and PRAS40 at threonine 246 in CLL samples as compared to normal donors (Figure 2A).

To help determine the specific molecular mechanisms that are altered in CLL, we interrogated the total proteins from the differentially expressed probes in the RPPA dataset and observed overrepresentation of signaling events regulating the PI3K/AKT pathway, with pathways specifically involved in the mRNA translation machinery of eukaryotic cells (Figure 2B, Supplementary File S2). Overrepresentation of these pathways biologically corresponds with the phosphorylation of the translation regulator 4E-BP1 at serine 65 and overexpression of eIF4G, AKT, mTOR, and SF2/ASF, a splicing protein recently implicated as a translational regulator [28, 50, 66, 67]. The upregulation of these protein alterations therefore highlights the possibility of mRNA translation as a tumorigenic mechanism in CLL (Figure 2C).

### 4E-BP1 serine 65 phosphorylation occurs independently of AKT phosphorylation in CLL

**Figure 3:**
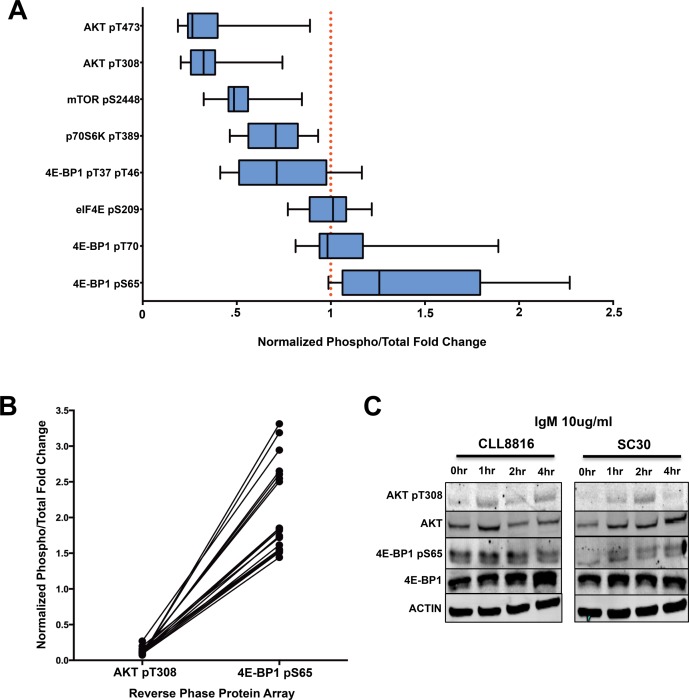
4E-BP1 serine 65 phosphorylation is independent of AKT/mTOR activation in CLL **A.** Normalized phosphorylation fold changes of AKT/mTOR substrates in CLL samples from RPPA (error bars = 95% CI). **B.** Paired comparison of CLL samples demonstrating the disconnection between AKT T308 phosphorylation and 4E-BP1 serine 65 phosphorylation. **C.** IgM activation of CLL sample 8816 and normal donor SC30. 4E-BP1 seems to be constitutively phosphorylated in CLL regardless of AKT pT308 activation, whereas the phosphorylation of 4E-BP1 in normal B-cells is dependent upon IgM-mediated AKT activation.

We then compared the distinct phosphorylation events of the AKT/mTOR pathway to reveal the overall phosphorylation patterns of AKT/mTOR substrates in CLL. By normalizing the phosphoprotein levels to the internal levels of the corresponding total protein, we observed 4E-BP1 at serine 65 had higher phosphorylation levels in CLL compared to healthy donors. However, the high phosphorylation at this site did not correspond with other AKT/mTOR related phosphorylation sites, including AKT threonine 308, AKT serine 473, p70S6K threonine 389, and mTOR serine 2448, which were all relatively hypophosphorylated (Figure 3A). These phosphorylation patterns are quite interesting due to the fact that 4E-BP1 phosphorylation typically is dependent upon AKT phosphorylation [68, 69].

Because these B-cells were obtained from the peripheral blood and significant phosphorylation of AKT typically occurs through microenvironment interactions in the lymph node, we wanted to determine how AKT activation compares with 4E-BP1 serine 65 phosphorylation during *ex vivo* stimulation. To accomplish this feat, we performed a side-by-side activation of CLL B-cells cells and healthy donor B-cells with 10ug/ml anti-IgM for 1, 2, and 4 hours to determine the precise AKT threonine 308/4E-BP1 serine 65 phosphorylation pattern in CLL. Based on our results, AKT phosphorylation was higher in both CLL and normal B-cells during IgM stimulation. However, where healthy donors demonstrated increased phosphorylation of 4E-BP1 during IgM stimulation, CLL cells demonstrated consistently high and unchanging levels of 4E-BP1 phosphorylation, regardless of AKT activation (Figure 3C). Based on the results, it seems 4E-BP1 serine 65 phosphorylation in normal B-cells is dependent upon AKT phosphorylation, whereas 4E-BP1 sustains a constant phosphorylated state independent of AKT activation in CLL B-cells. This result would suggest that a disconnect takes place between AKT activation and 4E-BP1 phosphorylation during CLL transformation.

### Increased CLL apoptosis corresponds with eIF4G cleavage and decreased 4E-BP1 serine 65 phosphorylation

With the gained understanding of 4E-BP1 phosphorylation and eIF4G overexpression in CLL, we wanted to test whether targeting the effects of these proteins would affect CLL viability and survival. With this rationale in mind, we compared the inhibitory capability of the recently developed BTK inhibitor Ibrutinib and the PI3Kδ inhibitor Idelalisib against the NVP-BEZ235, a dual PI3K/mTOR kinase inhibitor that has demonstrated ability to attenuate both 4E-BP1 phosphorylation and eIF4G activity in AML [23, 69-71].

**Figure 4:**
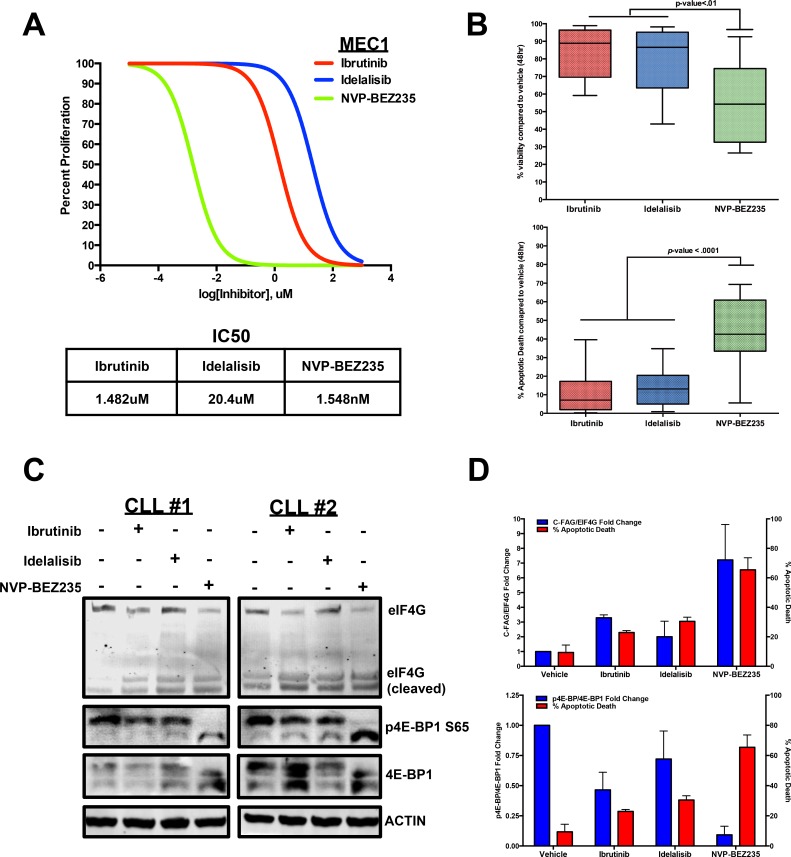
CLL apoptosis corresponds with eIF4G cleavage and 4E-BP1 dephosphorylation **A.** Dose-dependent treatment analysis of Ibrutinib, Idelalisib, and NVP-BEZ235 in the MEC1 CLL cell line to compare the IC50 for each inhibitor. **B.** Annexin V/DAPI apoptotic assay comparing 10uM Ibrutinib, 10uM Idelalisib, and 10uM NVP-BEZ235 treatment in CLL cells co-cultured with Huh7.5 cells (*n* = 15). Based on the results, NVP-BEZ235 was able to cause more apoptotic death during the 48hr period. **C.** At 6 hours, 10uM NVP-BEZ235 causes greater dephosphorylation of 4E-BP1 as well as greater cleavage of eIF4G in HS-5 co-cultured CLL cells compared to 10 uM Ibrutinib and 10uM Idelalisib. **D.** Decreasing 4E-BP1 phosphorylation and increasing eIF4G cleavage correspond with higher apoptotic death in primary CLL samples (errors bars = S.E.M.).

To compare these three inhibitors, we first performed an IC50 proliferation assay for each of the three inhibitors in the MEC1 CLL cell line. From this experiment, we determined NVP-BEZ235 to have a lower IC50 value (Figure 4A). We then co-cultured 15 CLL patient B-cells with Huh7.5 cells, an adenocarcinoma cell line that provides a proper microenvironment for survival and viability of CLL cells [64], and treated the primary cells with each inhibitor for 48 hours at their maximum effective doses of 10uM [23, 70, 72]. Based on Annexin V/DAPI measurements, NVP-BEZ235 was able to significantly lower cell viability compared to Ibrutinib and Idelalisib. This lowered viability correlated with a potent induction of apoptosis in CLL cells as NVP-BEZ235 caused a significant increase in Annexin V+/DAPI+ cell death compared with side-by-side treatment of Ibrutinib and Idelalisib (Figure 4B).

We then wanted to determine if any differential 4E-BP1 and eIF4G alterations occurred when treating CLL cells with the three inhibitors. To observe any differential effects, we co-cultured CLL cells with HS-5 stromal cells and performed a side-by-side treatment with Ibrutinib, Idelalisib, and NVP-BEZ235. After 6 hours, we observed little distinction in AKT phosphorylation (T308 and S473) and AKT-dependent mTOR S2448 phosphorylation when comparing the three treatments (Supplementary Figure S1). However, we did observe a significant difference in 4E-BP1 phosphorylation when comparing the three treatments as NVP-BEZ235 significantly diminished 4E-BP1 serine 65 phosphorylation compared to Ibrutinib and Idelalisib, both of which caused little change in 4E-BP1 phosphorylation (Figure 4C).

Interestingly, this decrease in 4E-BP1 phosphorylation also correlated with greater cleavage of the eIF4G protein. This event previously characterized in immortalized lymphoma cell lines treated with chemotherapy [65], currently has not been characterized in clinical CLL samples. However, by treating CLL patient B-cells with the 3 inhibitors, we observed the lowest levels of the 220kDa eIF4G expression with NVP-BEZ235 treatment, while simultaneously observing increased detection of the protein fragments that correspond in size with the previously identified C-terminal fragment of apoptotic cleavage of eIF4G (C-FAG) (Figure 4C) [73, 74]. This observation implies that the mRNA translation is ultimately disrupted during apoptotic induction due to the enhanced apoptotic cleaving of the eIF4G translation initiator protein. Finally, we collectively compared the semi-quantitative 4E-BP1 phosphorylation and eIF4G cleavage levels with the Annexin V+/DAPI+ cells in each treatment and determined that greater apoptotic induction corresponds with greater 4E-BP1 dephosphorylation and eIF4G cleavage, suggesting that attenuating the protein events that upregulate translational initiation can impede cell survival mechanisms in CLL (Figure 4D).

## DISCUSSION

By conducting RPPA analysis on primary CLL samples using 167 different antibody probes, we have been able to provide a comprehensive view of specific protein signaling and expression changes that take place in CLL. Specifically, we were able to determine from our selected samples that CLL contains a common protein expression signature that is seemingly more consistent among the CLL subtypes characterized by different genetic abnormalities. This common protein signature coincides with previous results demonstrating general overexpression of BCR-related signaling proteins, like BTK, regardless of a patient's clinical subtype [21, 23, 24].

By further analyzing our clinical RPPA dataset through differential expression analysis between CLL and healthy B-cells, we revealed upregulation of proteins that are commonly associated with CLL pathogenesis, including apoptotic resistance (e.g. BCL2 upregulation, BAX downregulation, cleaved caspase-7 downregulation) and PI3K/AKT/mTOR pathway proteins (e.g. AKT, mTOR, IRS1, GAB2, p70S6K) [29, 31, 36, 39], while also identifying overexpression of oncogenic proteins that are not well classified in CLL (e.g. BRAF, STAT5A, DVL3). It is also interesting to note that upregulation of certain proteins were somewhat counterintuitive, specifically TSC1, TSC2, and PDCD4 as these proteins are typically associated with tumor suppression [75, 76]. However, these proteins are also negatively regulated by phosphorylation, and their phosphorylation states are not known based on our array study. Nevertheless, the differential protein expression patterns discovered in our RPPA samples provide further clarity and insight into how certain molecular mechanisms are altered in CLL.

Understanding the expression and activation states of numerous proteins simultaneously allows for better knowledge of how to target these oncogenic events in CLL. Within the realm of the AKT/mTOR pathway, we specifically determined that proteins involved in mRNA translational regulation were being upregulated in CLL patient samples, revealing a potential characteristic of CLL oncogenesis. These aberrant events regarding protein translation include overexpression of the translation initiator eIF4G and phosphorylation of the cap-dependent translation regulator 4E-BP1, an event directly regulated by mTOR activity [68]. These two events have previously been identified as drivers of other cancer types, like breast cancer and AML, yet neither event currently has been described as oncogenic factors in CLL [28, 69].

Nevertheless, the intriguing aspect of this translational machinery upregulation was its uncoupling from BCR-mediated AKT activation. Though total AKT and mTOR are upregulated in our RPPA dataset, the PI3K/AKT-related phosphorylation activity for these proteins appears to be negligible. This type of result is expected as the RPPA CLL patient samples were collected from the peripheral blood and are not in their active state, which occurs in the lymph node. However, 4E-BP1 serine 65 phosphorylation, an event that typically is regulated by the cascading phosphorylation of the PI3K/AKT/mTOR pathway [68], was hyperphosphorylated regardless of AKT activation as demonstrated by IgM stimulation comparisons. This result would suggest that 4E-BP1 phosphorylation, a typical indicator of translational activation, is potentially needed to help maintain survival of dormant CLL cells in the peripheral blood.

Currently, we do not know the specific mechanisms that mediate sustained 4E-BP1 phosphorylation in the absence of AKT activation. One possibility could be the dysregulation of phosphatases that mediate dephosphorylation of 4E-BP1. One particular phosphatase, PPM1G, has previously been identified as a 4E-BP1-specific phosphatase [77]. However, investigation into publically available gene expression and mutation datasets suggests that PPM1G is not highly altered in CLL patients [4, 78]. Nevertheless, dysregulation of other phosphatases could play a significant role in sustaining 4E-BP1 phosphorylation in CLL. However, we did observe in our RPPA dataset overexpression of the splicing protein SF2/ASF. SF2/ASF has previously been identified as an oncogenic mediator of mTOR-related translation activation in the absence of AKT activation [66, 79, 80]. Specifically, this protein has been shown to facilitate the phosphorylation of 4E-BP1, potentially by scaffolding together the cap-dependent mRNA translation complex and mTOR complex 1 [67]. Therefore, SF2/ASF may potentially mediate phosphorylation of 4E-BP1 in CLL B-cells even in the absence of AKT phosphorylation. Nevertheless, further studies are still required for determining the precise mechanisms that maintain 4E-BP1 phosphorylation in CLL.

This potential disconnect between AKT activation and 4E-BP1 activation provided rationale for investigating the effectiveness of simultaneously targeting both upstream and downstream of the PI3K/AKT/mTOR pathway in CLL. We examined this concept by determining the inhibitory capability of the dual PI3K/mTOR inhibitor NVP-BEZ235. From our results, we saw that 4E-BP1 and eIF4G were significantly altered in CLL cells treated with NVP-BEZ235 when compared to Ibrutinib and Idelalisib, two drugs that target upstream BCR-related effectors involved in PI3K/AKT activation [20, 21]. The inhibitory results of NVP-BEZ235 in CLL correspond with the effectiveness of NVP-BEZ235 treatment in other AKT/mTOR-driven cancer types, as well as with previous data demonstrating suppression of eIF4G activity and 4E-BP1 phosphorylation in AML [69, 71].

Thus, with the coordinating results of our RPPA and inhibitor treatment comparisons in CLL patient samples, we demonstrate a rationale for considering mTOR-mediated mRNA translation as a therapeutic target in CLL. This general idea of targeting the mTOR pathway with rapamycin-based inhibitors has previously been investigated in CLL. However, current understanding of the rapamycin-induced negative feedback loop, which causes increased AKT signaling, provides explanation of why treating with single-agent rapalogs may have been ineffective in the clinical setting [81, 82]. More recent evidence has demonstrated the effectiveness of using the eIF4-mimetic 4EG-1 as a means to sensitize CLL cells towards apoptosis in pre-clinical studies, subsequently underlining the critical role of mRNA translation in CLL pathogenesis [83]. Based on our corresponding results that demonstrate the overexpression of eIF4G, the phosphorylation of 4E-BP1, and the subsequent reversal of both events in NVP-BEZ235-mediated CLL apoptosis, we provide further insight into the potential interplay between mRNA translation activation and CLL survival as well as demonstrate a proper strategy for effectively targeting this aberrant molecular mechanism in CLL.

## MATERIALS AND METHODS

### Reverse Phase Protein Array & Immunoblotting

Peripheral blood samples from CLL patients were obtained with patient consent and approved for use by the Institutional Review Board of Georgia Regents University. Peripheral blood samples from normal donors were purchased from a local blood bank. Once blood samples were obtained, CD19+ B-cells were isolated using the RosetteSep^TM^ B-cell isolation kit (STEMCELL Technologies, Vancouver, Canada). The isolated B-cells were then prepared in RIPA buffer supplemented with proteasome inhibitor and phosphatase inhibitor (Pierce, Rockford, IL, USA). The lysates were prepared to provide 1-1.5mg/ml of total protein lysate. RPPA analysis of the prepared lysates was then performed as previously described [49]. For this particular study, 118 total and 49 phospho-specific antibodies were used to determine expression and phosphorylation levels of the corresponding proteins (list of antibodies in Supplementary File S1). Validation immunoblotting for selected probes was performed by running 30ug of B-cell lysates on 4-20% gradient polyacrylamide gels (Bio-Rad, Hercules, CA, USA), transferred onto PVDF membranes (EMD Millipore, Billerica, MA, USA), and probed using corresponding primary antibodies from the RPPA study. Primary antibodies were detected with either an anti-mouse 680 red fluorescent antibody or an ant-rabbit 800 green fluorescent antibody (Pierce, Rockford, IL, USA). Immunoblot detection was performed using an LI-COR Odyssey (LI-COR Biosciences, Lincoln, NE, USA).

### Cell culture, reagents, and kinase inhibitors

All cell culture experiments were maintained in RPM1640 Medium with 1% Penn/Strep (Hyclone, Waltham, MA, USA) and 10% Human Serum (Mediatech, Manassas, VA, USA). For co-culture experiments, primary CLL cells were seeded at 1×10^7^ cells per well of a 6-well dish and co-cultured with either Huh7.5 cells or HS-5 cells at a density of 1×10^6^ cells per well of a 6-well dish. NVP-BEZ235, Ibrutinib (PCI-32765), and Idelalisib (GS-1101) were obtained from Selleck Biochem (Houston, TX, USA). Inhibitors were reconstituted in DMSO in order to treat primary CLL cells at a 10uM concentration.

### Cell proliferation, cell viability and apoptosis

Cell proliferation of the MEC1 CLL cell line was measured using the PrestoBlue Cell Viability reagent (Invitrogen, Waltham, MA, USA). Briefly, 25,000 MEC1 cells were treated with Ibrutinib, Idelalisib, or NVP-BEZ235 for 72 hours with a log-fold increase in concentration for each inhibitor. After 72 hours, MEC1 cells were incubated with PrestoBlue reagent for 2 hours and measured on a Spectramax Plus fluorescence plate reader (Molecular Devices, Sunnyvale, CA, USA) in order to determine an IC50 for each inhibitor in MEC1.

Cell viability and apoptosis of inhibitor-treated primary CLL cells was assessed by Annexin V+/DAPI apoptotic assay using an Annexin V-FITC antibody (Biolegend, San Diego, CA, USA) and DAPI staining (Sigma-Aldrich, St. Louis, MO, USA). Annexin V and DAPI staining of primary cells were measured using an LSRII flow cytometer (Becton Dickinson, Franklin Lakes, NJ, USA).

### Statistical analysis

Hierarchical clustering analysis for RPPA was performed using Partek Genomics Suite 6.6 (St. Louis, MO, USA) and pathway analysis was performed using Ingenuity Pathway Analysis (Qiagen, Hilden, Germany). Statistical measures for individual protein analysis and apoptotic studies were assessed using GraphPad Prism 6 (GraphPad Software, La Jolla, CA, USA).

## SUPPLEMENTARY FIGURE AND TABLES






