# Supplementary material for: RPPA-based protein profiling reveals eIF4G overexpression and 4E-BP1 serine 65 phosphorylation as molecular events that correspond with a pro-survival phenotype in chronic lymphocytic leukemia
Source: Oncotarget. 2015 May 12;6(16):14632–45. doi: 10.18632/oncotarget.4104 (PMC4546493; doi:10.18632/oncotarget.4104)
Supplement: Supplementary file 1 [file oncotarget-06-14632-s001.pdf]

# RPPA-based protein profiling reveals eIF4G overexpression and 4E-BP1 serine 65 phosphorylation as molecular events that correspond with a pro-survival phenotype in chronic lymphocytic leukemia

**Supplementary Material**

**Supplementary File S1: Normalized RPPA intensities of each analyzed antibody probe from CLL and normal B-cell samples.**

**Supplementary File S2: Ingenuity pathway analysis of proteins significantly altered between CLL and normal B-cells.**

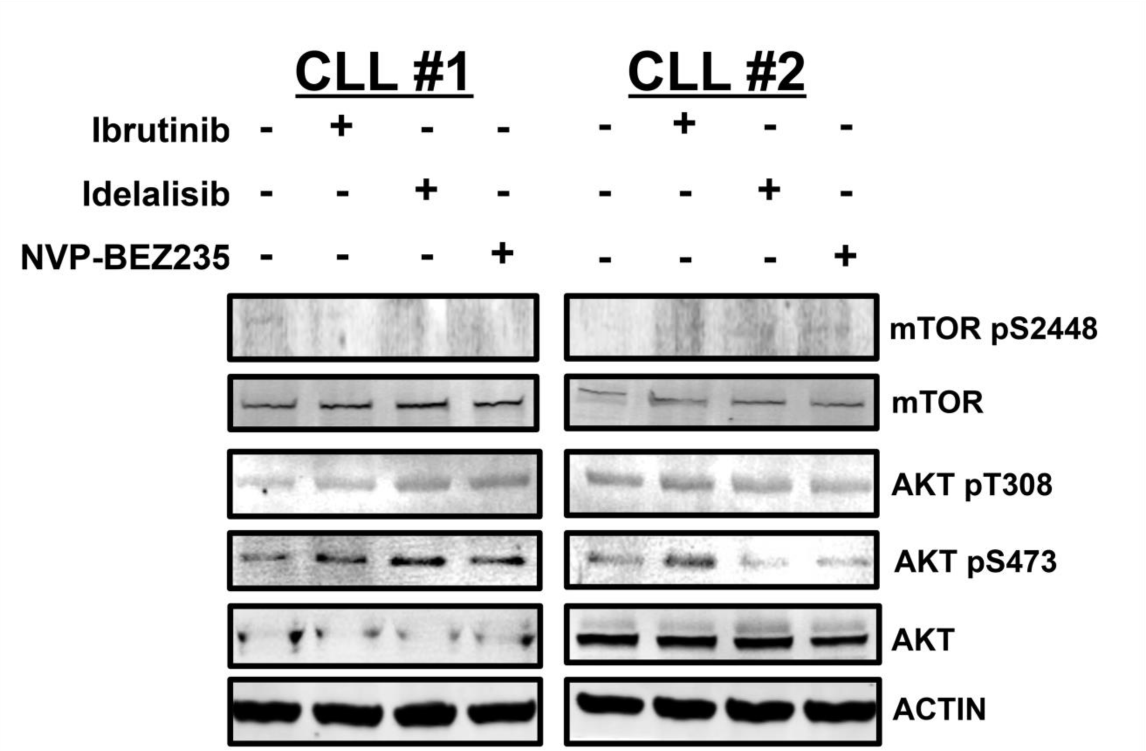


**Supplementary Figure S1: Comparison of AKT and mTOR phosphorylation in inhibitor treated CLL cells co-cultured with HS-5 stromal cells.**
